# Developing a statewide infection prevention program assessment service for dialysis settings using a six-sigma framework

**DOI:** 10.1017/ash.2023.309

**Published:** 2023-09-29

**Authors:** Chelsea Ludington, Renee Brum

## Abstract

**Background:** Due to the need for recurrent and direct access to the bloodstream, patients who require hemodialysis are at higher risk of developing healthcare-associated infections. Failure to assess gaps in systems and processes impedes the implementation of quality and performance improvement initiatives. In Michigan, there is no consultative service offered to dialysis units to assist with infection prevention practices, and no statewide dialysis data are being utilized. The Michigan Department of Health and Human Services developed a consultative, nonregulatory service dedicated to providing a comprehensive assessment of dialysis-based infection prevention programs. **Methods:** A multidisciplinary team created an infection prevention dialysis evaluation program using the six-sigma define–measure–analyze–design–verify model. These elements included content within the dialysis-specific Infection Control Assessment and Response (ICAR) Tool from the CDC with supporting program assessment items. From August 2021 through August 2022, the team completed 17 inpatient dialysis assessments within our cohort’s 17 hospitals. Data were analyzed using descriptive statistical analysis, and the final analysis included 1,086 observations from the developed assessment tool. Observations were grouped into 7 infection prevention categories: appropriate use of single and multiuse devices and supplies, aseptic technique, bloodborne pathogen prevention, cleaning and disinfection, hand hygiene, personal protection equipment (PPE) use, and storage of devices and supplies. Detailed summary reports were provided to the participating facilities after each site visit that included identified gaps, recommendations for improvement, and evidence-based resources. **Results:** Deficiencies were grouped into 7 major infection prevention categories among the 17 assessments, including cleaning and disinfection (n = 17, 100%), hand hygiene (n = 9, 53%), PPE use (n = 9, 53%), appropriate use of single and multiuse devices and supplies (n = 6, 35%), bloodborne pathogen prevention measures (n = 6, 35%), aseptic technique (n = 5, 29%), and storage of devices and supplies (n = 4, 24%). **Conclusions:** Our program’s prototype has been successful at detecting gaps in dialysis-based IP programs. By conducting data analyses of assessment findings, we have been able to assist the organization in establishing priorities for quality and performance improvement. Based on the results, comprehensive and robust systems to assess infection prevention programs, including those in dialysis settings, are necessary to enhance infection prevention operations across the continuum of care.

**Disclosures:** None

